# Hydatid Cyst in the Thigh: An Unusual Extra-hepatic Site

**DOI:** 10.7759/cureus.67929

**Published:** 2024-08-27

**Authors:** Varun Shetty, Saurav Shetty K, Iqbal M Ali

**Affiliations:** 1 General Surgery, Dr. D. Y. Patil Medical College, Hospital and Research Centre, Dr. D. Y. Patil Vidyapeeth, Pune (Deemed to Be University), Pune, IND

**Keywords:** rare, pair, vastus lateralis, thigh, hydatid cyst

## Abstract

*Echinococcus granulosus*, a cystic parasite, is the cause of hydatid illnesses. The liver and lungs are the most commonly affected organs. In this study, we report a rare instance of a hydatid cyst of the thigh. A 42-year-old male had been experiencing swelling on his left thigh that gradually increased in size. Upon examination, a firm, non-tender swelling measuring 12 x 8 x 8 cm at the lateral aspect of the left thigh was present. The diagnosis of a hydatid cyst in the thigh was confirmed by magnetic resonance imaging (MRI). The patient underwent surgical intervention and had an uneventful recovery post-operatively. The treatment for a hydatid cyst in the thigh, a rare parasitic condition that presents as a painless swelling and is typically diagnosed through MRI, involves en bloc resection. Therefore, surgeons must consider hydatidosis as a potential diagnosis when evaluating swelling in the thigh.

## Introduction

*Echinococcus granulosus*, a cestode from the *Taeniidae* family, is the primary cause of endemic cystic parasite infestations collectively labeled as hydatid diseases (HDs). These parasites are commonly found among farm workers who handle livestock daily and are involved in agriculture [1]. The tapeworm has intermediate hosts, including cattle and sheep as well as definitive hosts such as dogs [2]. Humans, on the other hand, are accidental hosts to acquire the infection. The liver, lungs, and brain are most often affected due to their rich blood supplies [3]. However, it is rare for the musculoskeletal system to be affected by these parasites. Even in areas where the disease is common, primary hydatid cysts in the musculoskeletal system are uncommon [4]. These cases can easily be mistaken for soft tissue sarcomas due to their ambiguous clinical presentations. We present the case of a 42-year-old male patient who sought medical attention for persistent thigh swelling, which was misdiagnosed as a thigh sarcoma at a primary healthcare center, illustrating the challenges faced and the treatment protocols used.

## Case presentation

A 42-year-old army officer visited the surgical outpatient department of our tertiary healthcare center with a six-month history of persistent, progressively enlarging swelling in the right thigh. He also reported cramping pain over the swelling while walking. During previous visits to two primary healthcare facilities, he had received conservative treatment with pain relief medications following a misdiagnosis. There was no history of a sudden increase in the size of swelling, trauma, or similar lesions in the past. Additionally, he did not report any fever, chills, or difficulties at work.

A general physical examination was unremarkable. However, a thorough clinical evaluation revealed a 12 × 9 × 7 cm firm and tender swelling on the outer aspect of the middle third of the right thigh, with pain over the swelling, aggravated especially while walking. The skin over the swelling was normal, and there was no restriction in the range of movements.

**Figure 1:**
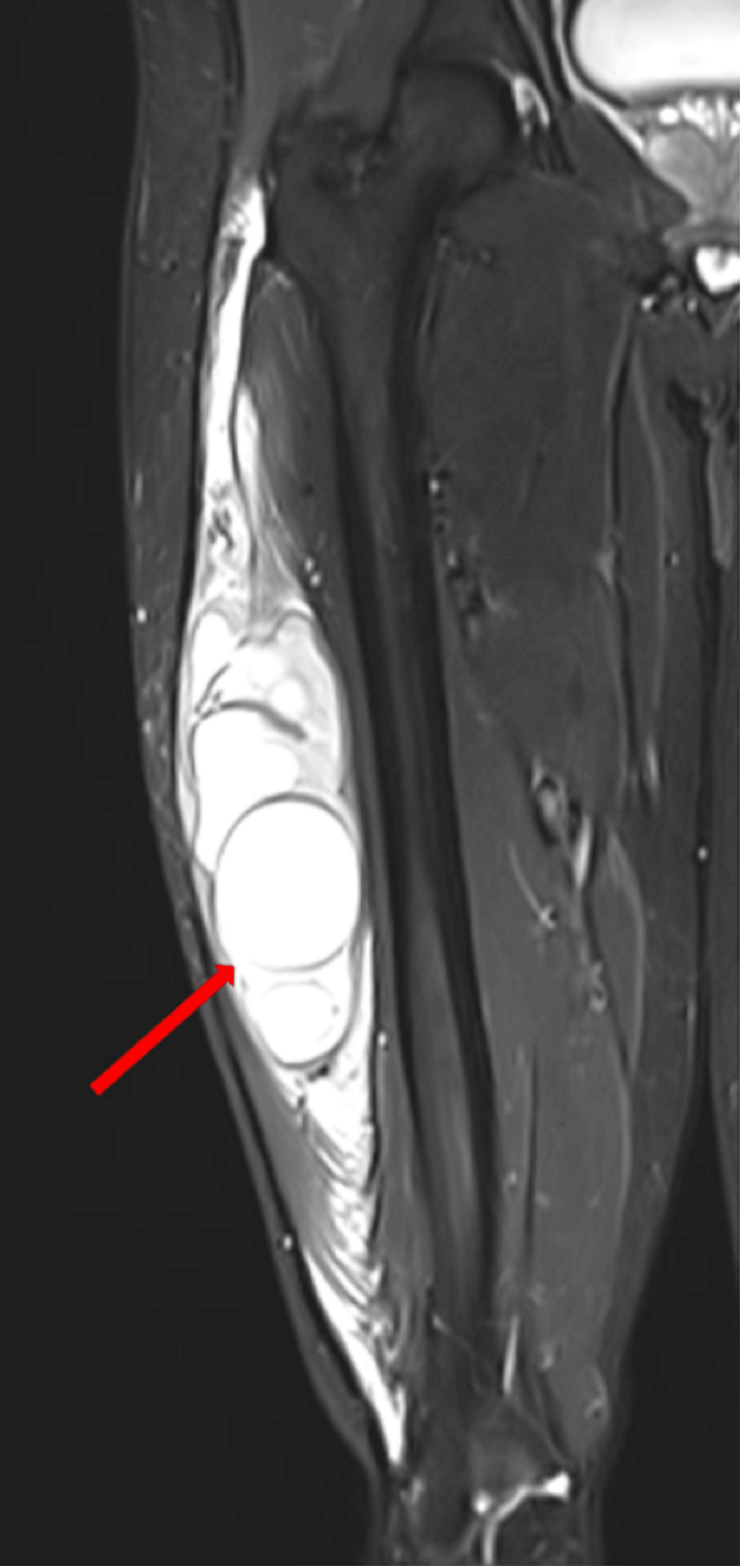
STIR coronal image of the right thigh Large cystic lesion with multiple daughter cysts involving the right vastus lateralis muscle, accompanied by surrounding muscle and subcutaneous edema. STIR: short tau inversion recovery

**Figure 2:**
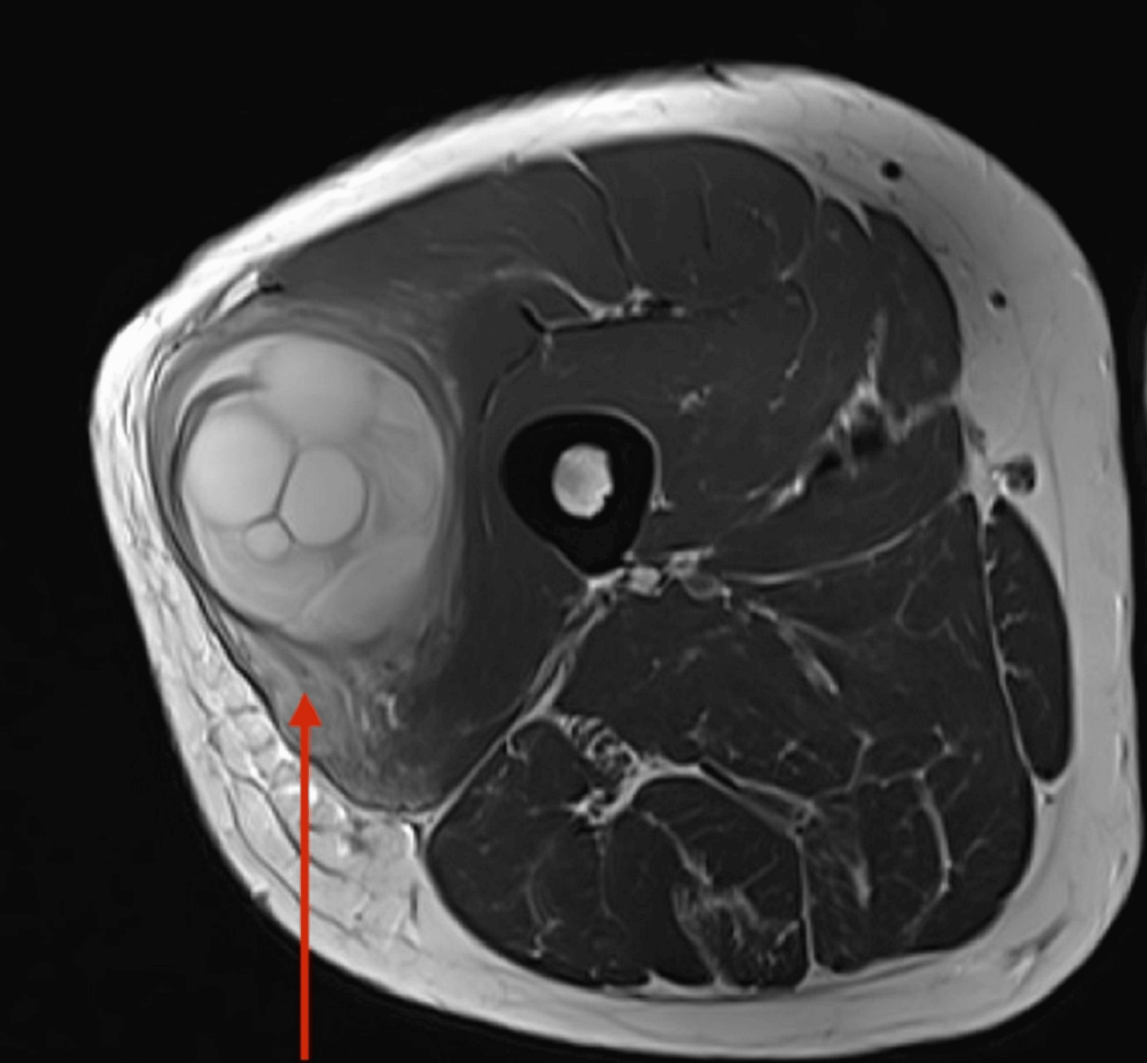
T2WI image of the right thigh Large cystic lesion with multiple daughter cysts involving the right vastus lateralis muscle, accompanied by surrounding muscle and subcutaneous edema. T2WI: T2 weighted imaging

An X-ray of the right thigh (AP and lateral views) showed no bone involvement, with the swelling confined to the muscular compartment. Ultrasonography (USG) of the right thigh indicated a 12 × 8 × 8 cm complex, multiloculated cystic lesion in the middle third of the right thigh, involving the vastus lateralis muscle belly. Magnetic resonance imaging (MRI) of the right thigh revealed a large cystic lesion with multiple internal daughter cysts, measuring 13.5 × 6.7 × 5.1 cm, involving the vastus lateralis muscle (Figure 1). The lesion appeared hypointense on T1 weighted image (T1WI) and hyperintense on T2WI images (Figure 2). Serpiginous foci, specifically seen in hydatidosis, were noted within the mass.

**Figure 3:**
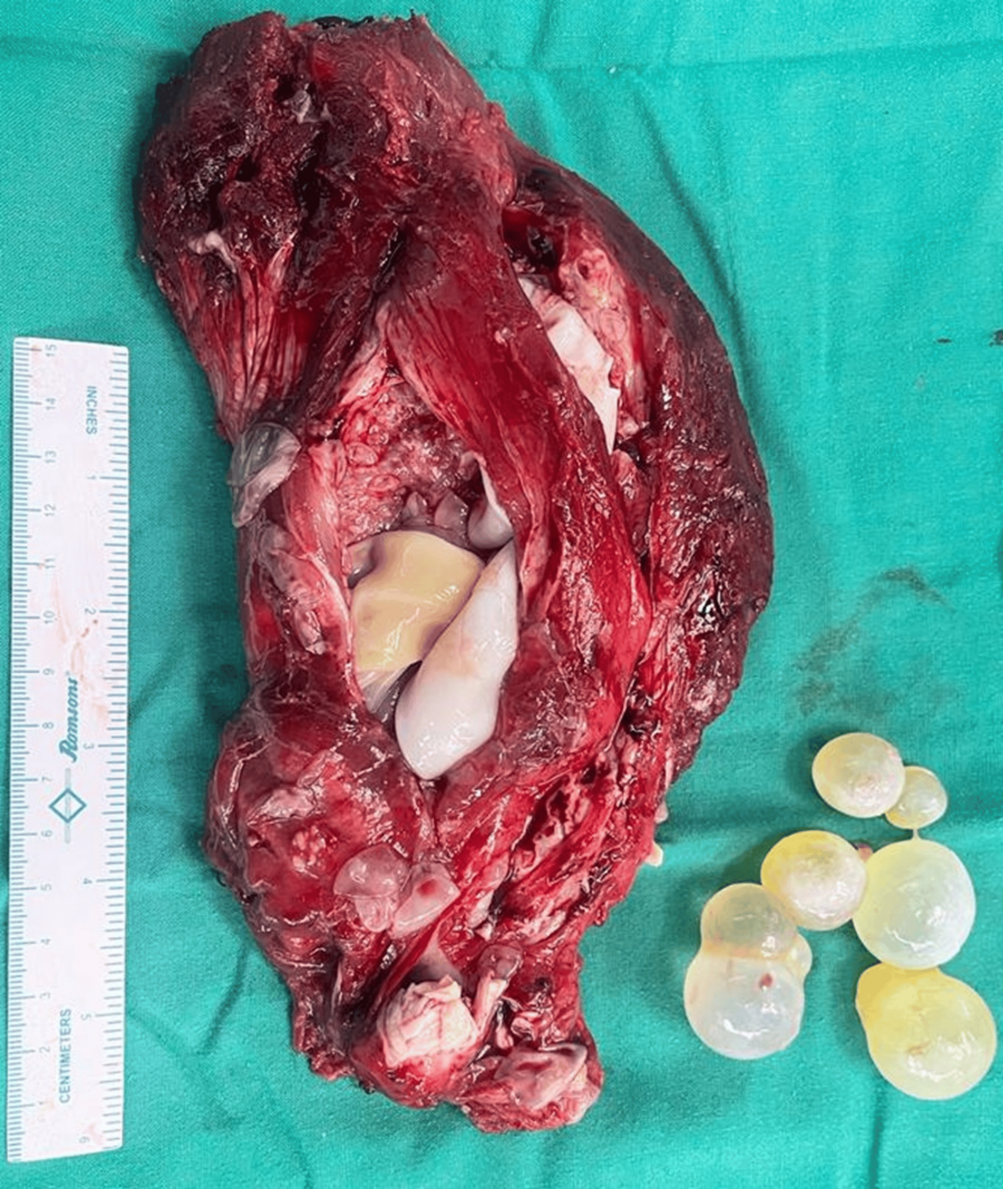
Excised specimen of the vastus lateralis muscle belly, showing the hydatid cyst cavity within.

A chest X-ray and ultrasound of the abdomen and pelvis revealed no additional hydatidosis foci in the body. Consequently, an excision of the cystic lesion along with the belly of the vastus lateralis muscle was planned, using all precautions to avoid spillage of contents and resultant anaphylaxis. The procedure was uneventful, and the patient had a smooth postoperative recovery. Upon opening the specimen, multiple daughter cysts were identified (Figure 3). Histopathology confirmed the diagnosis, and the patient was discharged on postoperative day 14 with anti-helminthic medications for three months. At a follow-up visit three months later, the patient was symptom-free.

## Discussion

*E. granulosus*, once primarily an endemic zoonotic disease, is now widespread due to rapid advancements in global travel. It is most prevalent in countries where sheep farming is common and within India, southern states including Tamil Nadu and Andhra Pradesh have been shown to report most cases [5,6]. HD is known to manifest either as primary or secondary infections. Transmitted through the ingestion of eggs, primary HD can affect nearly any organ. The most commonly affected organs are the lungs and liver. The spread of larvae from the initial site to other organs leads to the development of secondary infestation and may involve the liver, lungs, or spleen [7].

Manifestation of this disease in intramuscular tissue is rare due to constant contraction and lactic acid in the tissue. The patients affected exhibit painless and slow-expanding mass with normal overlaying skin [7]. These cysts grow gradually and can become space-occupying lesions, exerting pressure on surrounding tissues [8].

There is ongoing debate about the pathogenesis of muscle localization. Some suggest it results from direct implantation via a wound, such as a dog bite, while most experts believe that after passing through the colon, the embryo enters the muscles through systemic circulation [9]. To minimize the risk of disease transmission and allergic reactions, incisional biopsies and marginal excisions are contraindicated if a hydatid cyst is suspected, as the cystic fluid is highly harmful and contains significant foreign proteins [9,10].

Accurate clinical and radiological assessment is crucial to identify any additional primary sites. Imaging techniques like computed tomography (CT), USG, and MRI provide diagnostics leads by describing the location and characteristics of the cyst. The growth stage, tissue involved and complications caused determine the imaging features of a hydatid cyst ranging from cystic lesions to solid appearing masses [10]. USG is effective for diagnosis, providing detailed images of cystic membranes, septa, hydatid sand, floating membranes, daughter cysts, and vesicles. CT can reveal the internal structure of the cyst, cyst infection, and wall calcification, though calcification is uncommon in skeletal muscle cysts, and daughter cysts, frequently seen in hepatic hydatidosis, are rarely observed [10].

MRI is the preferred test if intramuscular HD is suspected, as it can effectively visualize most symptoms of the disease, except for calcifications. Typically, the primary cyst contains several smaller daughter cysts [8,10]. In our case, intramuscular hydatidosis was diagnosed preoperatively based on MRI findings. In addition, MRI helps to differentiate HD from other differentials such as soft tissue sarcoma. Pre-operative identification or suspicion of a hydatid cyst is crucial to prevent the rupture and systemic leakage of cyst contents during resection, which could cause allergic reactions. The current treatment approach involves complete excision of the cyst. Pre-operative treatment with oral anti-helminthic agents such as albendazole/mebendazole can help to reduce intra-cystic pressure, thereby reducing the risk of rupture and contamination during surgery. To prevent recurrence, a thorough irrigation of the cyst bed with hypertonic saline is done. Systemic antiparasitic medications are advised to complement these procedures. Post-surgery, ongoing clinical evaluation is necessary to monitor for recurrence.

In India, intramuscular hydatidosis as a manifestation of endemic HD is extremely rare. Therefore, diagnosing is challenging and requires a multi-modal approach. Among the various differential diagnoses for thigh swellings, from soft tissue sarcomas to traumatic hematomas, hydatidosis should be considered when determining the management plan. As demonstrated in our case, a combination of surgical intervention and medical treatment is essential to provide optimal care and prevent recurrence.

## Conclusions

Primary intramuscular hydatidosis is difficult to diagnose due to its rarity and similarity with other conditions like soft tissue sarcomas and traumatic hematomas. Due to the rarity and possibility of misdiagnosis, clinicians should consider HD in the differential diagnosis for thigh swellings, especially in endemic regions. Diagnostic accuracy depends on the combination of clinical evaluation and imaging techniques. As discussed in this case, early identification and appropriate surgical intervention, along with antiparasitic treatment, is crucial for effective management and prevention of recurrence.
